# Beating-heart surgical treatment of tricuspid valve papillary fibroelastoma: A case report

**DOI:** 10.1097/MD.0000000000004690

**Published:** 2016-08-26

**Authors:** Weidong Li, Junnan Zheng, Hengchi Zhao, Hongfei Xu, Yiming Ni

**Affiliations:** Department of Thoracic and Cardiovascular Surgery, First Affiliated Hospital of Zhejiang University, School of Medicine, Hangzhou, China.

**Keywords:** cardiac surgery, cardiac tumor, case report, papillary fibroelastoma, tricuspid valve

## Abstract

**Background:**

Cardiac papillary fibroelastomas are rare. And only 15% of the papillary fibroelastomas are located on tricuspid valve. However, the treatment of papillary fibroelastomas varies.

**Case summary:**

We report a 75-year-old Chinese male who was hospitalized because of a right atrial mass found by echocardiography. Complete tumor excision along with Kay's tricuspid valvuloplasty surgery on beating heart under cardiopulmonary bypass was performed to the patient. Pathologic examination confirmed the definite diagnosis of cardiac papillary fibroelastoma. The recovery of the patient was uneventful and echocardiographic examination performed 6 months after surgery revealed no recurrence of the tumor.

**Conclusions:**

Beating-heart surgical excision is an effective and safe treatment of tricuspid papillary fibroelastomas.

## 1 Introduction

Primary cardiac tumors are very rare. Their frequency in autopsy studies are below 0.03%^[1,2]^ and represent only 0.4%^[3]^ to 0.8%^[1]^ of all open-heart operations. Papillary fibroelastomas (PFEs) are the third most common primary tumor of the heart^[4]^ and they most commonly affect cardiac valves.^[5]^ Approximately 15% of the PFEs are located on the tricuspid valve.^[6]^ Although usually considered asymptomatic, PFEs can be associated with transient ischemic attack, stroke, myocardial infarction, sudden death, heart failure, presyncope, syncope, pulmonary embolism, blindness, and peripheral embolism.^[6]^ Surgical resection of the tumor has been proved safe and curative.^[6]^

Here we present the diagnostic evaluation and successful surgical resection on beating heart of a cardiac tumor, which was found on a 75-year-old Chinese patient with a recent ischemic stroke history. Histopathological result revealed papillary fibroelastoma at tricuspid annulus.

## 2 Case report

A 75-year-old Chinese man was referred to our local for surgical treatment of a right atrial mass. The mass was incidentally found on echocardiography when the patient was being treated for acute ischemic stroke 1 month ago. His past medical history was insignificant except for hypertension.

**Figure 1:**
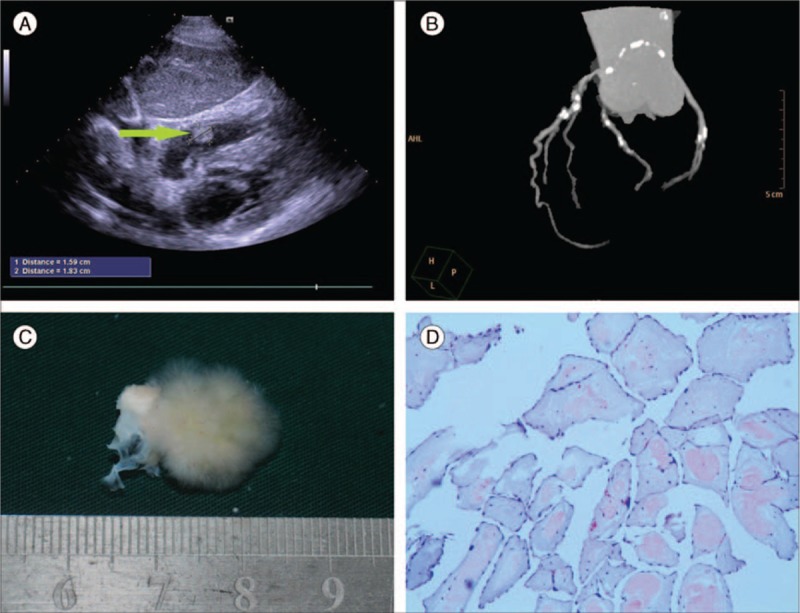
(A) Preoperative transthoracic echocardiogram showing a highly mobile mass in the right atrium near the tricuspid annulus. The arrow indicates the mass, 1.83 cm × 1.59 cm large. (B) Coronary CT angiography revealed mild to moderate stenosis and multiple calcified plaques of all main coronary arteries (left main, left anterior descending, left circumflex, and right coronary artery). The CT angiography showed right coronary artery dominance. (C) Gross view of the tumor showing a gelatinous mass resembled a sea anemone, ∼2 cm × 1.5 cm large, with a nodular stalk. (D) Histologic section of the tumor showing an avascular central core of hyalinized connective tissue surrounded by a connective matrix, covered by a single layer of endocardial cells.

On admission, a physical examination did not reveal any significant abnormalities. Electrocardiography revealed normal sinus rhythm and all laboratory blood results were unremarkable. Carotid ultrasound was performed and did not show obvious carotid artery stenosis or atherosclerotic plaques. Transthoracic echocardiography (TTE) demonstrated a normal left ventricular ejection fraction and a slight aortic valve regurgitation. The other heart valves appeared unremarkable. However, a small mobile nodular tumor mass at the right atrium was seen (Fig. 1A). Subsequent coronary computed tomography angiography (CTA) revealed mild to moderate stenosis and multiple calcified plaques of all main coronary arteries (left main, left anterior descending, left circumflex, and right coronary artery) (Fig. 1B).

Perioperative transesophageal echocardiography (TEE) at the time of surgery demonstrated a small spherical, pedunculated mass (1.8 cm × 1.7 cm) near the tricuspid annulus in the right atrium. The right atrium was not enlarged and the tricuspid valve appeared complete and functional. The patient underwent surgery through a median sternotomy under cardiopulmonary bypass established via aorto-bicaval cannulation. The heart was not arrested. When the right atrium was opened, the tumor was found close to the interatrial septum. The short stalk of the tumor arose from the septal portion of the tricuspid annulus. Complete tumor excision on beating heart was then performed. Then, a Kay's tricuspid valvuloplasty on the beating heart was performed by placing a double pledget-supported mattress suture of 2-0 Ethibond from the anteroposterior commissure to the posteroseptal commissure along the posterior annulus and obliterated the posterior leaflet. Repeated intraoperative TEE showed no residual mass, no septal defect, and no tricuspid regurgitation. The patient was weaned from bypass without difficulty and the recovery was uneventful. The patient was extubated within 12 hours after surgery and discharged home on postoperative day 7. A 6-month follow-up after surgery with echocardiogram showed no recurrence of the tumor and no tricuspid regurgitation.

On gross examination, the tumor was ∼2 cm × 1.5 cm large, and the stalk was nodular. When viewed under physiologic saline, it has a frond-like appearance and resembled a sea anemone (Fig. 1C). Histopathological examination of the tumor showed a stalk with multiple radiating papillary fronds. These villous fronds consisted of a central core of acellular collagen covered by a layer of endocardial cells (Fig. 1D). Thus, the definite diagnosis of cardiac papillary fibroelastoma was made.

## 3 Discussion

Papillary fibroelastoma is a rare cardiac tumor, representing <10% of primary tumors.^[7]^ Most frequently, PFEs are found on the cardiac valves (90%) and the tricuspid valve has been shown an localization for only 17% of PFEs.^[8]^ However, it has become increasingly apparent that tumors arising from tricuspid valve can sometimes cause cardiac-related symptoms such as chest discomfort, exertional dyspnea, palpitation or cerebrovascular accidents due to tricuspid incompetency, arrhythmia, intermittent right ventricular outflow partial obstruction, and pulmonary embolism.^[9]^

Most cases of PFEs were found incidentally while the patients were being evaluated for an unrelated problem or physical finding. Transthoracic echocardiography is usually the initial diagnostic method in patients with suspected cardiac mass. PFEs may appear speckled with echolucencies and a stippled pattern near the edges on echocardiography but were generally well-demarcated and homogenous in appearance.^[10]^ However, echocardiography was not always precise in diagnosing PFEs. Study had shown that the sensitivity and specificity of TTE were both <90%, with an overall accuracy of 88.4% for the detection of PFEs > 0.2 cm.^[11]^ Other diagnostic measures mainly included chest roentgenograms, CT scan, and magnetic resonance imaging (MRI). Chest x ray may reveal cardiac chamber enlargement or pulmonary hypertension related to mitral valve PFEs. Also, calcification of PFEs can be seen on radiographic examination. Although PFEs are most often difficult to identify on cardiac CT due to small size and mobility, they may appear hypodense with irregular borders attached by a thin stalk and mobile on cine ECG-gated CT imaging if visible.^[12]^ MRI, especially Gadolinium enhanced, can typically demonstrate a PFE mass on a valve leaflet or on the endocardial surface.^[6]^ Further a cine gradient-recalled echo MRI may demonstrate turbulence of blood flow caused by vavular PFEs.^[6]^

Considering the risk of serious embolic complications, surgical removal is recommended once these tumors have been detected.^[7]^ No or minor valve repair is usually possible when cardiac valves are involved.^[13]^ In our case, although the patient is 75 years old and had a recent stroke history, the tumor size (> 1 cm) along with the high chances of preserving the native valve at operation, led us to decide in favor of the surgical option.

Preoperative coronary CTA of the patient showed mild to moderate stenosis and multiple calcified plaques of all main coronary arteries (Fig. 1B). This might be related to the advanced age, hypertension, and atherosclerosis, which might also contributed to the previous cerebrovascular accident of the patient. However, considering the less-than-moderate stenosis of the coronary artery as well as the stroke history, coronary artery bypass grafting was not considered as concomitant procedure of the tumor resection surgery.

The use of cardioplegic arrest with aortic cross-clamping is the standard procedure for myocardial protection in cardiac tumor resection surgery. However, due to coronary artery stenosis and advanced age of the patient, we preferred to excise the fibroelastoma using the on-pump beating-heart technique. This strategy allowed better myocardial protection by avoiding cardioplegia and its ischemic consequences. Continuous perfusion of the heart without cross-clamping has been considered an old but effective technique of myocardial protection.^[14]^ Another advantage was the maintenance of the tricuspid valve competence just after the removal of the fibroelastoma.

## 4 Conclusions

We reported a rare case of papillary fibroelastoma arising from the tricuspid annulus, which was detected by echocardiography preoperatively and confirmed by histology afterward. Using on-pump beating-heart surgery, the tumor was resected completely along with tricuspid valvuloplasty.
